# Environmentally friendly loading of palladium nanoparticles on nanoporous PET track-etched membranes grafted by poly(1-vinyl-2-pyrrolidone) *via* RAFT polymerization for the photocatalytic degradation of metronidazole†

**DOI:** 10.1039/d3ra03226d

**Published:** 2023-06-20

**Authors:** Nursanat Parmanbek, S. Duygu Sütekin, Murat Barsbay, Nurgulim A. Aimanova, Anastassiya A. Mashentseva, Assel N. Alimkhanova, Alisher M. Zhumabayev, Alyona Yanevich, Alimzhan A. Almanov, Maxim V. Zdorovets

**Affiliations:** a The Institute of Nuclear Physics of the Republic of Kazakhstan 050032 Almaty Kazakhstan a.mashentseva@inp.kz; b Department of Chemistry, L.N. Gumilyov Eurasian National University 010008 Astana Kazakhstan; c Department of Chemistry, Hacettepe University 06800 Ankara Turkey; d Polymer Science and Technology Division, Institute of Science, Hacettepe University Beytepe 06800 Ankara Turkey; e Department of Nuclear Physics, New Materials and Technologies, L.N. Gumilyov Eurasian National University 010008 Astana Kazakhstan; f Engineering Profile Laboratory, L.N. Gumilyov Eurasian National University 010008 Astana Kazakhstan; g Department of Intelligent Information Technologies, The Ural Federal University 620002 Yekaterinburg Russia

## Abstract

Nanoporous track-etched membranes (TeMs) are highly versatile materials that have shown promise in various applications such as filtration, separation, adsorption, and catalysis due to their mechanical integrity and high surface area. The performance of TeMs as catalysts for removing toxic pollutants is greatly influenced by the pore diameter, density, and functionalization of the nanochannels. In this study, the synthesis of functionalized poly(ethylene terephthalate) (PET) TeMs with Pd nanoparticles (NPs) as catalysts for the photodegradation of the antibiotic metronidazole (MTZ) was methodically investigated and their catalytic activity under UV irradiation was compared. Before loading of the Pd NPs, the surface and nanopore walls of the PET TeMs were grafted by poly(1-vinyl-2-pyrrolidone) (PVP) *via* UV-initiated reversible addition fragmentation chain transfer (RAFT)-mediated graft copolymerization. The use of RAFT polymerization allowed for precise control over the degree of grafting and graft lengths within the nanochannels of PVP grafted PET TeMs (PVP-*g*-PET). Pd NPs were then loaded onto PVP-*g*-PET using several environmentally friendly reducing agents such as ascorbic acid, sodium borohydride and a plant extract. In addition, a conventional thermal reduction technique was also applied for the reduction of the Pd NPs. The grafting process created a surface with high-sorption capacity for MTZ and also high stabilizing effect for Pd NPs due to the functional PVP chains on the PET substrate. The structure and composition of the composite membranes were elucidated by scanning electron microscopy (SEM), X-ray diffraction (XRD) analysis, thermogravimetry, contact angle measurements and energy dispersive X-ray (EDX), X-ray photoelectron (XPS) and Fourier transform infra-red (FTIR) spectroscopies. The effects of different types of reducing agents, pH, the amount of loaded catalyst and MTZ concentration on the MTZ catalytic degradation efficiency of the obtained composites were investigated. The efficiency of the catalyst prepared in the presence of ascorbic acid was superior to the others (89.86% removal at 30 mg L^−1^ of MTZ). Maximum removal of MTZ was observed at the natural pH (6.5) of the MTZ solution at a concentration of 30 mg per L MTZ. The removal efficiency was decreased by increasing the catalyst dosage and the initial MTZ concentration. The reaction rate constant was reduced from 0.0144 to 0.0096 min^−1^ by increasing the MTZ concentration from 20 to 50 mg L^−1^. The photocatalyst revealed remarkable photocatalytic activity even after 10 consecutive cycles.

## Abstract 2

Poly(1-vinyl-2-pyrrolidone) functionalized PET TeMs with Pd nanoparticles provide high catalytic ability for the photodegradation of the metronidazole, and the highest degradation efficiency was obtained in the presence of Pd_Asc@PVP-*g*-PET membrane.

## Graphical abstract

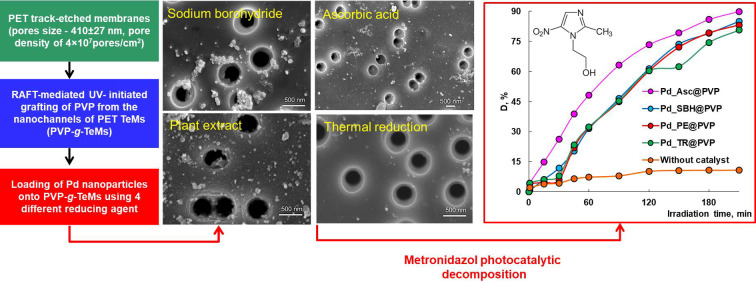

## 1. Introduction

Antibiotics have been used for many decades to prevent and treat bacterial infections and have played a crucial role in improving human health and increasing life expectancy.^1,2^ Antibiotics are medications that are specifically designed to kill or inhibit the growth of bacteria, and they work by targeting different parts of the bacterial cell, such as the cell membrane or DNA.^3^ Antibiotics are often used to treat bacterial infections in humans and animals. However, when these drugs are improperly disposed of during their manufacturing process or use, they can leak into environmental sources and have harmful effects on the ecosystem. Organic pollutants, including antibiotics like metronidazole, can accumulate in soil and water, leading to a variety of adverse environmental effects. For example, they can contribute to the growth of antibiotic-resistant bacteria, making it more difficult to treat infections in humans and animals.^4–6^ Therefore, it is important to minimize the release of these compounds into the environment and to manage and treat wastewater appropriately in order to protect the health of humans and animals, as well as aquatic ecosystems.^7,8^

In recent years, there has been a growing interest in the use of nanoporous photocatalysts for the degradation of antibiotics in aqueous media.^9–12^ This is due to the increasing concern about the environmental impact of antibiotics, which are commonly used in human and veterinary medicine, and their presence in wastewater. Nanoporous photocatalysts are materials that can harness the energy of light to drive chemical reactions, including the degradation of antibiotics. These materials typically have a large surface area, which allows for more efficient adsorption and interaction with the antibiotics.^13^ Membranes with controllable high roughness, such as nanoporous track-etched membranes (TeMs), are created by bombardment with accelerated heavy ions and subsequent chemical etching, resulting in a nanoporous structure with high aspect ratios and a rough surface. The controllable high roughness of TeMs makes them ideal for creating unclogged and effective nanochannels, which can be utilized for various applications, such as water purification,^14–19^ drug delivery,^20^ sensing^21–23^ and DNA sequencing,^24^ and can work locally at high rates and with high selectivity.

Irradiation of TeMs with high-energy particles causes the scission of polymer chains and the formation of cross-sectional nanochannels following their etching. The modification of these nanochannels by grafting with other polymers offers another versatile and promising field of application.^25–27^ Graft copolymerization is a commonly used method to modify the surface properties of materials.^28^ Conventional graft copolymerization techniques based on free radical polymerization lack the precise control over molecular weights and architectures of the grafted polymer chains. This can lead to a high degree of heterogeneity in the resulting graft copolymers, which can limit their properties and applications. To address these limitations, reversible-deactivation radical polymerization techniques, such as reversible addition–fragmentation chain transfer (RAFT) polymerization, have been successfully used in the synthesis of well-defined graft copolymers with controlled molecular weights and architectures.^29^ In RAFT polymerization, a chain transfer agent is used to control the polymerization process, allowing for precise control over the molecular weight and architecture of the resulting polymers. These agents also allow for graft copolymerization from a variety of substrates in a controlled manner, resulting in well-defined grafted surfaces.^30,31^

Metronidazole (MTZ) is an antibiotic medication used to treat bacterial and protozoal infections. It is classified as a ‘potentially hazardous’ substance due to its potential toxicity to humans and other living organisms.^32,33^ For the removal of pollutants, polymers are widely used in different designs as adsorbents and catalysts.^34^ Poly(1-vinyl-2-pyrrolidone) (PVP) is an effectively used polymer for the removal of pollutants such as heavy metals, antibiotics and dyes from wastewater by absorption.^35,36^ Some nanoparticles have also been reported to be effective in the degradation of MTZ. Metal oxide NPs such as titanium dioxide (TiO_2_),^37^ zinc oxide (ZnO),^38^ and iron oxide (Fe_3_O_4_),^39^ have been extensively studied for their ability to adsorb and degrade various pollutants including metronidazole. Zerovalent metal NPs such as iron NPs,^40^ copper NPs,^41^ palladium NPs^42^ have also been shown to be effective in removing metronidazole.

This paper presents an efficient and novel method to functionalize the surface and nanochannel interiors of PET track-etched membrane by grafting PVP *via* RAFT-mediated graft copolymerization and then loading Pd NPs *via* several environmentally friendly reducing methods. The PVP-grafted PET template absorbed metronidazole and served as a stabilizing substrate for the reduced Pd NPs. The composite membranes were eventually investigated for their photocatalytic activity in the degradation of metronidazole and offered significant potential for environmental remediation due to their mechanical stability, high performance, and environmentally sensitive production process.

## 2. Materials and methods

### 2.1. Materials

Benzophenone (BP), ethanol, sodium hydroxide (NaOH), hydrogen peroxide (H_2_O_2_), palladium chloride (PdCl_2_), poly (1-vinyl-2-pyrrolidone) (PVP), hydrochloric acid (HCI), ascorbic acid, sodium borohydride (NaBH_4_), 4-cyano-4-(phenylcarbonothioylthio) pentanoic acid (CPPA), and metronidazole (MTZ) were purchased from Sigma-Aldrich (Schnelldorf, Germany). PVP was passed through an acidic alumina column to remove the inhibitor. CPPA was utilized as a RAFT agent. All aqueous solutions were prepared using deionized water (18.2 MΩ cm^−1^, Aquilon D-301).

### 2.2. Irradiation and track-etching of PET films

To obtain track membranes, a PET Hostaphan® RNK film (“Mitsubishi Polyester Film”, Germany) was used. The film was irradiated with ^84^Kr^15+^ ions at a specific energy and fluency using a cyclotron (Cyclotron DC-60, Institute of Nuclear Physics of Kazakhstan). After irradiation, the film was etched in 2.2 M NaOH to attain PET TeMs with nanochannels of an average pore diameter of 410 ± 27 nm and a pore density of 4 × 10^7^ pores per cm^2^. Samples were kept in air at room temperature.

### 2.3. Grafting of PVP from the nanochannels of PET TeMs (PVP-*g*-TeMs)

The grafting of PVP was initiated through the activation of benzophenone (BP), a photoactive tethering reagent immobilized on the PET surface. In order to increase the amount of immobilized BP, the PET surface was oxidized first to increase the carboxyl group concentration. The oxidation of PET was carried out in 500 mM H_2_O_2_ (hydrogen peroxide) solution at pH 3 for a duration of 180 minutes under UV irradiation at a wavelength of 254 nm and a power of 190 W.^43^ The process followed by washing the samples with deionized water, air-drying them for 5 hours, and then quantifying the amount of –COOH groups using a titration method. The –COOH groups were found to be 18.17 ± 3.2 mmol cm^−2^ based on five measurements. To immobilize BP, the oxidized PET TeMs were soaked in a solution containing 5% BP in DMF (w/v) and kept in a shaking water bath at 150 rpm for 24 hours at room temperature. The resulting membranes were washed with water and ethanol, dried, and then used for grafting experiments.

Grafting of PET TeMs (with a surface of 2 cm^2^) was carried out in a 10 mL of aqueous polymerization solution containing various amounts of monomer, *N*-vinyl pyrrolidone, VP (10, 20, 30, 40, 50 w/v%) and xanthate-based RAFT agent, CPPA, for time periods ranging from 60 to 600 min ([VP]/[CPPA]) = 1/1000. Reaction mixtures were put into sealed flasks and degassed with argon for 5 min. For UV-assisted graft polymerization, samples in glass vials were settled under a UV lamp (15 W at 295 nm, Ultra-Vitalux 300 W, Osram, Augsburg, Germany) at a distance of 12 cm. After the predetermined grafting period, the films (PVP-*g*-TeMs) were washed with deionized water and ethanol periodically to constant weight. Degree of grafting (DG) was determined gravimetrically (Mettler Toledo, Columbus, OH, USA) using eqn (1) and calculated with an accuracy of ±0.05 mg using the formula.1
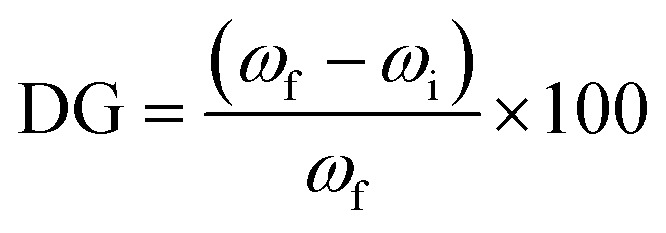
where, *ω*_f_ is the weight of the PVP-*g*-TeMs films and *ω*_i_ is the weight of the BP-immobilized PET TeMs.

### 2.4. Preparation of the plant extract

The extract of Betula Pendula Roth (BPR) was used as a plant-based reducing agent for Pd(ii) ions. The collection of BPR was carried out in mid-April 2022. The raw material collection area was in Meshchansky forest of the Petropavlovsk region (North Kazakhstan region). The dry raw materials (tree shoots) of BPR were crushed to a particle size of 2–3 mm, and the weight of the sample for each extraction experiment was 50 g. To obtain the maximum polyphenolic component extraction, a 96% (v/v) water–ethanol solution was used, the concentration of final plant extract of BPR tree shoots was 0.09 mg mL^−1^.

### 2.5. Loading of Pd NPs onto PVP-*g*-TeMs

Prior to loading of Pd NPs, PVP-*g*-TeMs were immersed in a saturated PdCl_2_ solution for 72 h in a shaker (130 rpm, IKA KS 3000 IS control, (IKA, Konigswinter, Germany)) at room temperature in order to achieve maximum Pb(ii) absorption. Afterwards, the absorbed Pd(ii) ions were reduced through 4 different methods, including the use of ascorbic acid,^44^ sodium borohydride,^45^ BPR plant extract and thermal treatment. The reduction time was 5 h for all the reducing methods. The standard reduction with sodium borohydride was performed at ambient temperature, while the rest at 70 °C. The amount of Pd NPs deposited was determined gravimetrically based on the difference in membrane mass before and after the reduction process with an accuracy of ±0.01 mg (AND BM-252G by AND, Japan) and expressed in units of mg cm^−2^.

### 2.6. Photocatalytic activity

Metronidazole (MTZ) was used as a model antibiotic to examine the photocatalytic activity of the prepared composite membranes under UV-light irradiation. All experiments were carried out in 200 mL double-wall glassware under UV-light using 15 W at 295 nm (Ultra-Vitalux 300 W, Osram, Augsburg, Germany). The distance from the light source to the solution was 15 cm. A 1 × 1 cm Pd_R@PET composite membrane was immersed in 20 mL of MTZ solution. R corresponds to the type of reducing agent, and is Asc, SBH, PE or TR, respectively, for the use of ascorbic acid, sodium borohydride, BPR plant extract and thermal reduction methods. The concentration of MTZ was ranged between 20 and 50 mg L^−1^ and the solutions were stirred in the dark for 15 min to achieve adsorption–desorption equilibrium between organic pollutant and catalyst. For analyzing photocatalytic activity, a 2.0 mL aliquot was taken every 15–30 min from the reaction mixture and its absorbance was measured using a Specord-250 spectrophotometer (Jena Analytic, Jena, Germany) in the wavelength range of 200–800 nm. According to the Beer–Lambert law, the concentration of MTZ is directly proportional to its absorbance. The degradation of MTZ was determined based on its characteristic peak at 320 nm using the following equation:2
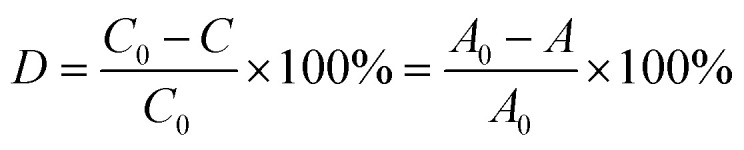
where *A*_0_ is the initial absorbance of MTZ solution at 320 nm before adding the catalyst membrane, *A* is the absorbance at 320 nm at different time intervals, and *C*_0_ is the concentration of the feed solution.

### 2.7. Characterizations

PET TeMs were visualized by environmental scanning electron microscopy (SEM, FEI Quanta 200F ESEM, ThermoFisher Scientific, Hillsboro, OR USA) operating at 10 kV. The elemental composition of the composites was studied by a Hitachi TM3030 SEM (Hitachi Ltd, Chiyoda, Tokyo, Japan) equipped with a Burker XFlash MIN SVE (Burker, Karlsruhe, Germany) microanalysis system at an accelerating voltage of 15 kV.

Attenuated Total Reflectance Fourier Transform Infra-Red (ATR-FTIR) spectroscopy measurements were carried out using a Spectrum One FTIR spectrometer (PerkinElmer, Waltham, MA, USA). Each spectrum was obtained from 32 scans in the wavenumber range of 4000–500 cm^−1^, with a resolution of 4.00 cm^−1^.

Diffuse reflectance DR-UV-Vis spectra of the synthesized composites were recorded at room temperature on a Analytik Jena Specord-250 spectrophotometer (Jena Analytic, Jena, Germany) with an integrating sphere for reflectance measurement.

The crystal structure of the nanoparticles was examined on a D8 Advance diffractometer (Burker, Karlsruhe, Germany) in the angular range of 2*θ* 30–80° with a step of 2*θ* = 0.02° (measuring time: 1 s, tube mode: 40 kV, 40 mA). The mean size of crystallites was determined *via* the broadening of X-ray diffraction reflections using the Scherer equation.^16^ The phase composition was determined using the Rietveld method, which is based on approximating the areas of the diffraction peaks and determining the convergence with reference values for each phase.^19^ The volume fraction of the composite phase was determined using equation:3
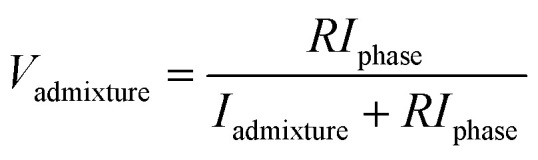
where *I*_phase_ is the average integral intensity of the main phase of the diffraction line, *I*_admixture_ is the average integral intensity of the additional phase, and *R* is the structural coefficient equal to 1.

XPS measurements were carried out using a Thermo Scientific K-Alpha spectrometer (Waltham, MA, USA) with a monochromatized Al Kα X-ray source (1486.6 eV photons) at a constant dwell time of 100 ms, pass energy of 30 eV with a step of 0.1 eV for core-level spectra and 200 eV with a step of 1.0 eV for survey spectra. The pressure in the analysis chamber was maintained at 2 × 10^−9^ Torr or lower. All samples were analyzed at a take-off angle of 90°. Surface elemental composition was determined using an X-ray spot size of 400 μm by varying the energy between 0 and 1000 eV.

The pore size of the pristine and grafted TeMs and the structural parameters of grafted composite membranes were determined by porometry using the Hagen–Poiseuille equation.^16^

Water contact angle (CA) values of oxidized and PVP grafted PET TeMs surfaces were measured at ambient temperature using a DSA-100 goniometer system (Krüss Company, Hamburg, Germany). The average CA was obtained by at least three repetitive measurements using 10 μL DI water and calculated by applying the Young–Laplace method of the Drop Shape Analysis program (Krüss Company, Hamburg, Germany).

Thermal properties of polymers were recorded using a PerkinElmer thermogravimetric analyzer Pyris 1 TGA (PerkinElmer, Waltham, MA, USA). Analyses were conducted over the temperature range from 40 to 800 °C with a programmed temperature increment of 25 °C min^−1^ under N_2_ atmosphere.

## 3. Results and discussions

### 3.1. Characterization of the composite membranes

Among the reversible-deactivation radical polymerization techniques allowing for the precise control of polymer architecture by temporarily deactivating or modifying the polymerization process, RAFT has emerged as a particularly versatile and widely used method for the controlled synthesis of complex polymeric structures. The success of the RAFT mechanism in controlling the polymer architecture depends on several key parameters including the selection of the RAFT agent. The RAFT polymerization of PVP can be successfully carried out using CPPA.^46^ BP is a photoactive tethering reagent that is commonly used to functionalize the surface of various materials, including commercial plastics and fabrics.^47^ It contains two aromatic rings linked by a carbonyl group (C=O), which makes BP a versatile molecule for surface modification through photochemistry. When exposed to UV-light, BP undergoes a photoreaction that results in the formation of highly reactive benzophenone triplet state. These species can then react with nearby polymer substrate surface to yield radicals, thereafter, creating a covalently tethered grafted layer in the presence of a monomer. The incorporation of functional species such as carboxyl groups on the surface of PET TeMs is reportedly increase the amount of immobilized BP moieties, and thus the degree of grafting in the next step.^48^

**Fig. 1:**
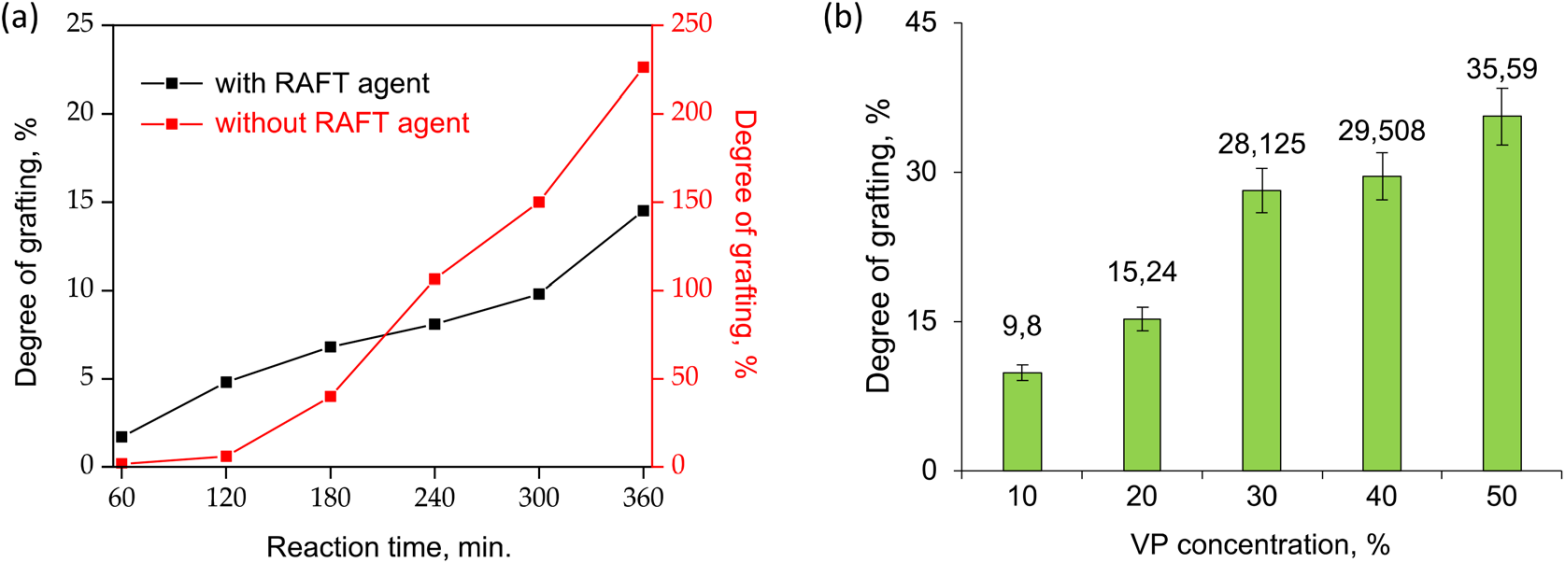
(a) Effect of reaction time on the UV-initiated RAFT-mediated and conventional grafting of PVP from BP-immobilized PET-TEMs in water, [VP]/[CPPA] = 1000 for RAFT-mediated grafting, monomer concentration = 20% (a), effect of monomer (VP) concentration on the UV-initiated RAFT-mediated grafting of PVP from BP-immobilized PET-TEMs in water, [VP]/[CPPA] = 1000, *t* = 360 min (b).

UV-induced PVP grafting was carried out by varying some reaction parameters, including monomer concentration and reaction time. The reaction time was varied from 30 minutes to 360 minutes in order to obtain the optimal polymerization time as summarized in Fig. 1a. Mass increase due to grafting was observed after about 60 min of UV-irradiation. As seen in Fig. 1a, the degree of grafting (DG) varied between 1.7% and 14.5% at 20% VP concentration. Extending the polymerization time further increases the degree of grafting, *i.e.*, 20% DG is achieved after 420 minutes of irradiation. However, the membranes became fragile after over 420 minutes of irradiation. For this reason, although the degree of grafting has increased, the reaction time has not been extended further since the decrease in mechanical strength may cause problems in repeated or long-term use.

To determine the effect of monomer concentration, irradiations were carried out at different monomer concentrations for 360 minutes. As seen in Fig. 1b, degree of grafting increased with increasing monomer concentrations in water (DG ranged from 9.8% to 35.6% for 10% to 50% VP concentrations). As expected, grafting degrees gradually increased with increasing VP concentration, with the highest DG being approximately 35% in the solution with 50% VP concentration. However, at the DGs above about 20%, the nanochannels were clogged, as can be seen from the SEM results to be discussed below. In addition, the membranes lose their flexibility and become more fragile at DGs exceeding roughly 20%. Therefore, 360 minutes reaction time and 20% (v/v) monomer concentration were chosen as the optimum conditions for further studies, in which the desired grafting level (approximately 15%) can be obtained without causing degradation of the PET template and closure of the nanochannels under UV irradiation.

Conventional grafting experiments carried out in the absence of RAFT agent resulted in much higher DG values compared to that obtained by RAFT mechanism under the same experimental conditions (Fig. 1a). The samples in which the conventional method was applied were over-grafted, visible heterogeneities were formed on the surfaces, the nanochannels were completely closed and the films were almost deformed. These results clearly indicate that grafting of PVP from PET TeMs proceeds in a controlled manner by the RAFT agent used, namely CPPA.

**Fig. 2:**
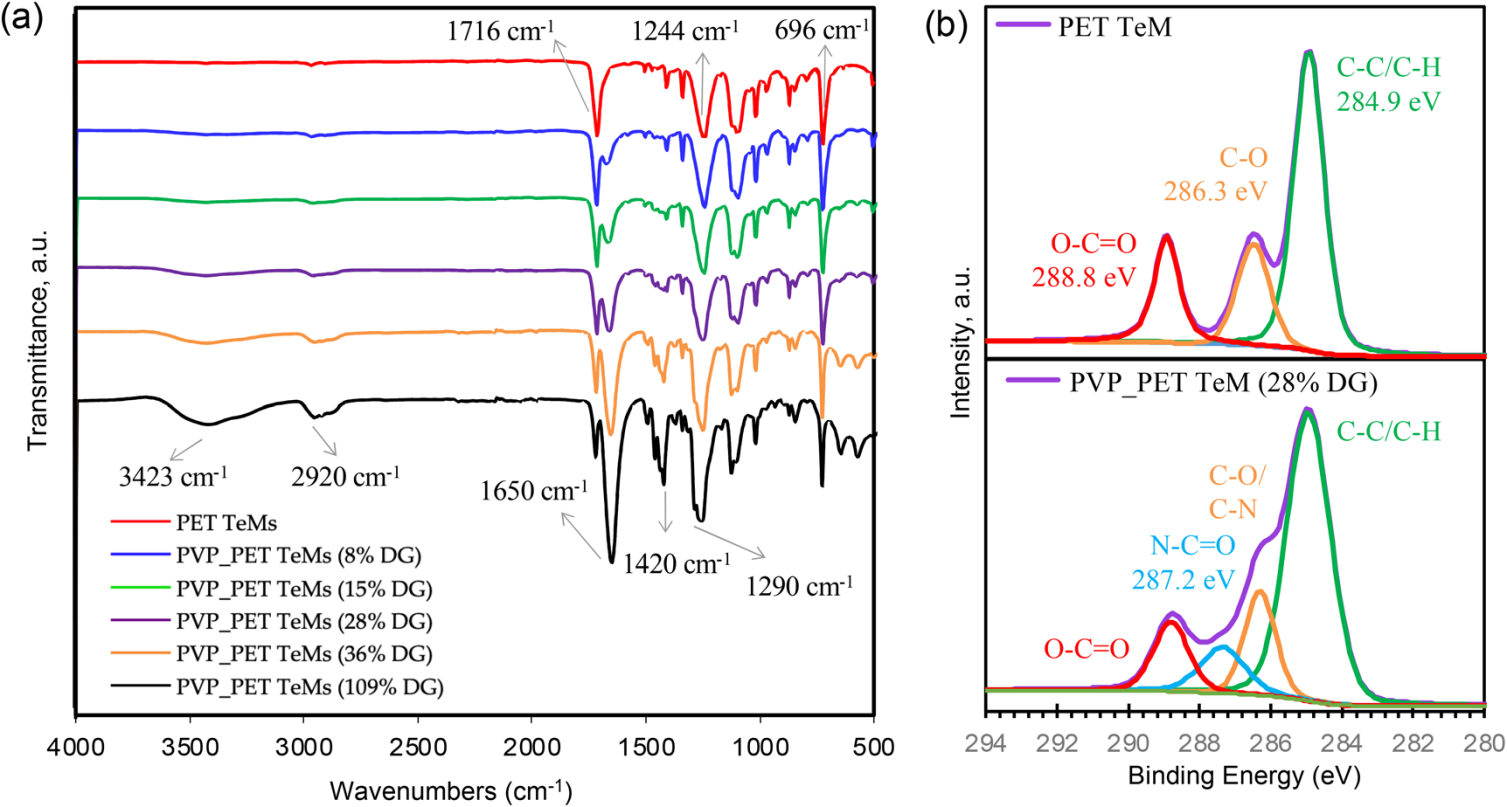
FTIR spectra of pristine PET TEM and PVP grafted membranes with different degrees of grafting (a), C 1s core level scans of pristine PET TeM (upper spectrum) and PVP grafted PET TeMs with 28% DG (b).

The chemical structures of pristine and PVP grafted PET TeMs with different DGs were evaluated using FTIR spectroscopy as shown in Fig. 2. Characteristic peaks of PET substrate were observed at 1716 cm^−1^, 1244 cm^−1^ and 696 cm^−1^ for C=O stretching of carboxylic ester group, C–C–O stretching of ester group, and out-of-plane C–H bending of aromatic ring, respectively. The grafting of PVP from the PET surface significantly gives rise to new peaks arising from PVP structure, such as the stretching vibration of O–H at around 3423 cm^−1^, C–H asymmetric stretching at 2920 cm^−1^, the stretching vibration of C=O at 1650 cm^−1^, C–H_2_ bending peaks around 1490–1420 cm^−1^ and C–N stretching at 1290–1020 cm^−1^. All these characteristic peaks of PVP become more pronounced with the increase in DG.

In order to elucidate the composition and chemical environment of the samples along the surface extending from the top monolayer to a depth of about 10 nm, XPS analysis was conducted. The C 1s spectrum of pristine PET TeM presented in the upper panel in Fig. 2b shows the binding energies corresponding to C–C, C–O and O–C=O species at 284.9 eV, 286.3 eV and 288.8 eV, respectively, thus confirming the structure of the TeM substrate.^49^ With the addition of PVP to the membrane composition after grafting, a new peak corresponding to the binding energy of C in N–C=O groups appeared at 287.2 eV in the spectrum of PVP-*g*-PET membrane with 28% DG. In addition, the peak at about 286.2 eV belonging to the C–N groups overlapped with the existing C–O peak and caused the relative amount of this peak to increase compared to the O–C=O peak of PET at 288.8 eV. Grafting of PVP chains having the C–C backbone broadened the C–C peak at 284.9 eV and increased its relative intensity. All these spectral changes confirm the grafting of PVP from the PET substrate.

**Fig. 3:**
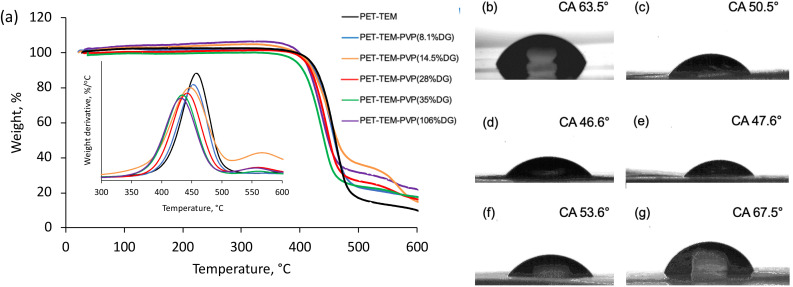
Thermal degradation profiles and the derivative thermogravimetry curves (inset) of pristine PET TeM and PVP grafted PET TeMs with different degrees of grafting (a), contact angle measurements of pristine PET TeM (b) and PVP grafted PET TeMs with different degrees of grafting: 8.1% DG (c); 14.5% DG (d); 28% DG (e); 35.6% DG (f); 106.39% DG (g).

Thermal characteristics of PET TeMs with different DGs were investigated using thermogravimetric analysis. The degradation profile of pristine PET TeMs present a characteristic one-step thermal degradation between 340 and 530 °C with a mass loss of 90.3% at 600 °C.^50^ The derivative thermogram suggests that (see inset of Fig. 3a), the maximum weight loss rate of pristine PET-TEMs occurred at 463 °C. Structural degradation of PVP occurs between 400–480 °C and exhibits a degradation profile similar to that of PET-TEMs. The weight loss at 600 °C was calculated as 82.9%, 82.5%, 82.8%, 82.6%, 78.1% for PVP grafted PET TeMs with DGs of 8.1%, 14.5%, 28%, 35%, 106%, respectively. This suggests that thermal stability of the grafted membrane is increased with DG. The thermal degradation of PVP and PET overlaps to a large extent, but the degradation of PVP occurs at a relatively lower temperature than PET.^51,52^ The derivative thermograms in the inset of Fig. 3a clearly show that with increasing DG values, the degradation peak of PVP grafted PET TEMs shifted to lower temperatures, confirming the increased amount of PVP in the graft copolymer structure.

**Fig. 4:**
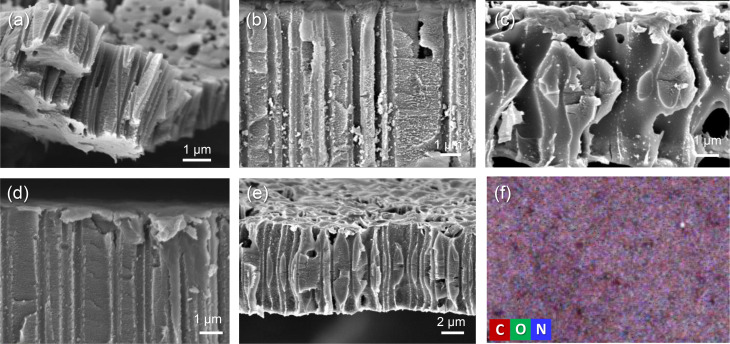
SEM Images of pristine PET TeM (a) and PVP grafted PET TeMs with different degrees of grafting: 8.1% DG (b); 14.5% DG (c); 28% DG (d); 35.6% DG (e) and SEM-EDX elemental mapping PVP-*g*-TeM (DG:14.5%) (f).

Contact angle (CA) measurement was carried out to investigate the wettability changes of the surface as a result of grafting of PVP. Pristine PET TeMs gave a contact angle of around 63.5° (Fig. 3b). After grafting, it was observed that the contact angle significantly decreased from 63.5° to 50.5° (Fig. 3c) for the sample with a DG of 8.1% and decreased further to 46.6° (Fig. 3d) for the sample with 14.5% PVP, presenting the hydrophilic character of PVP. However, after a DG of 35.6%, it was observed that the contact angle improved gradually with increasing of grafting degree (Fig. 3e–g). This may be attributed to the closure of the nanochannels as a result of excessive grafting of PVP, and the consequent increase in surface roughness.^53^ In order to investigate this phenomenon, SEM measurements were performed. For the pristine PET-TeMs, the pore diameter was about 410 ± 27 nm (Fig. 4a), while the diameter of the PVP grafted membrane with 14.5% DG was about 318 ± 14 nm (Fig. 4b), highlighting a 90 nm reduction in the diameter of the nanopores. SEM images of other membranes also revealed that the pore diameter decreased as a function of DG, assessing gradual saturation of nanochannels and reflecting the living/well-controlled character of RAFT-mediated radical copolymerization between PVP grafts and the PET TeMs (Fig. 4a–e). Fig. 4d shows that at a degree of grafting of 28%, the surface of the membrane is covered with a thin layer of PVP and the nanochannel entrances are blocked. The SEM-EDX atomic mapping image presented in Fig. 4f for C, O, and N illustrated that these elements were uniformly distributed over the entire surface at a DG of 14.5%, and the surface was homogeneously covered with PVP, mainly due to the presence and distribution of N atoms. As the immobilized BP can cleave the labile hydrogen atoms only on the topmost surface level, the radicals generated are on the surface and thus the growing of PVP chains only occurs from the surface, not through the inside the PET bulk.

**Fig. 5:**
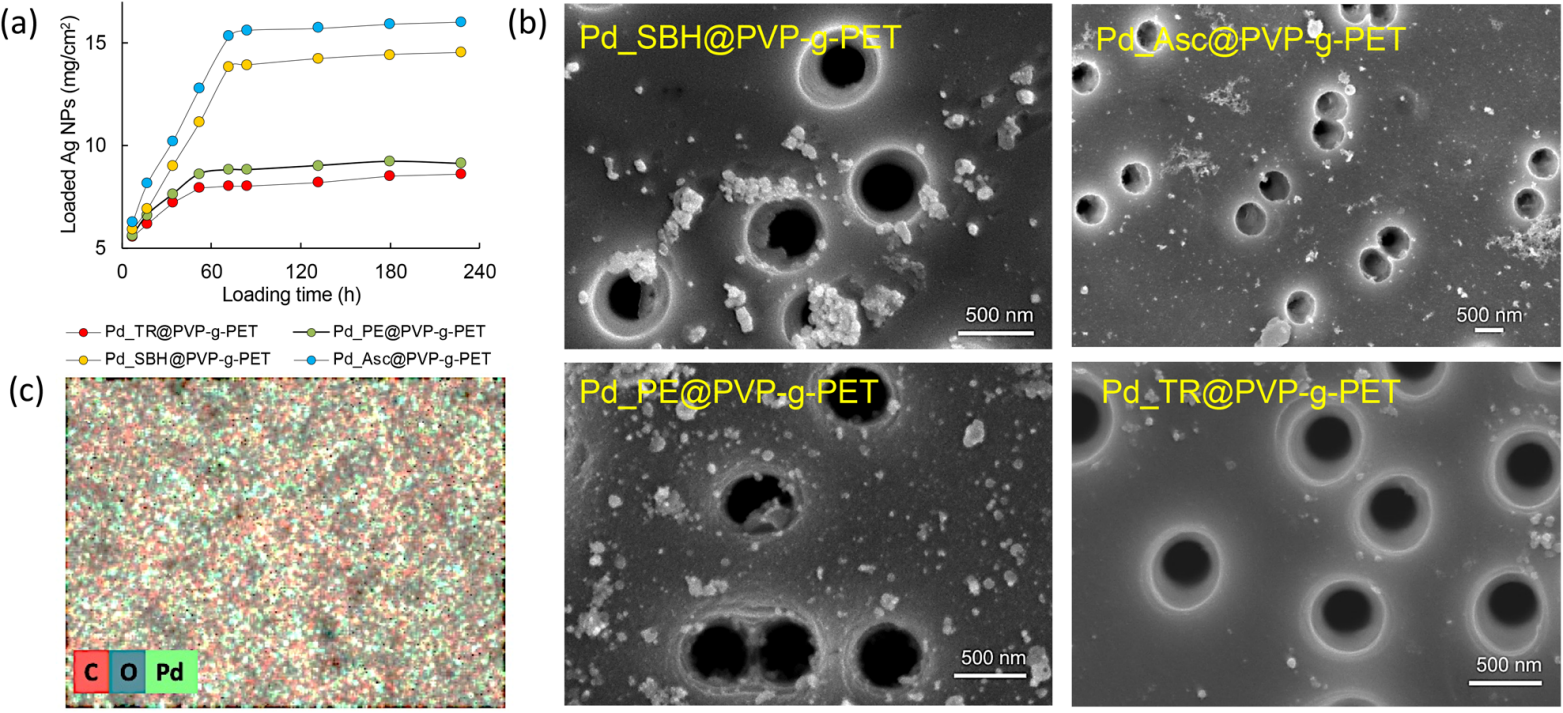
Pd NPs loading efficiency onto PVP-*g*-TeM (DG: 14.5%) for 4 different reducing methods applied by using ascorbic acid (Asc), sodium borohydride (SBH), BPR plant extract (PE) and thermal (TR) reduction (a), SEM images of PVP-*g*-TeMs loaded with Pd NPs obtained by different reducing methods (b). SEM-EDX surface elemental mapping of Pd_Asc@PVP-*g*-PET (c).

It is well known that PVP acts as a stabilizer promoting the formation of Pd NPs and plays a crucial role in the catalytic performance of metal NPs.^54^ The reduction of Pd(ii) ions to Pd(0) metal NPs were performed using four different techniques and their effectiveness were compared. As depicted in Fig. 5a, the amount of loaded Pd NPs can be easily increased by varying the reaction time. On the other hand, it was observed that the reduction method plays an important role in the amount of loaded NPs. Ascorbic acid (Asc) illustrated more desirable results as a reducing agent than the other methods during the adsorption. Sodium borohydride also yielded a considerable amount of stabilized Pd NPs. In contrast, using the thermal and plant extract mediated reduction methods, it was found that the extract was slightly superior to thermal reduction in terms of loaded NPs amount, but both resulted in lower degrees of loading than the other two methods. The reaction reached equilibrium after approximately 72 hours of loading in all methods, and the highest amount of loaded Pd NPs was found to be around 16 mg cm^−2^ for the reduction carried out using ascorbic acid.

Photographs of the membranes before and after Pd NPs loading by different techniques are shown in Fig. S1 (ESI†). The darkening in the images is due to the loaded Pd NPs. The photographs also revealed that the tendency of color change obtained in the application of different reducing methods is consistent with the observations in Fig. 5a. The surface morphology of PVP grafted PET-TeMs loaded with Pd NPs was examined by scanning electron microscopy (Fig. 5b). For all Pd@PVP-*g*-PET membranes, it was observed that the deposition of Pd NPs was achieved without closing the nanopores of the polymer template. Moreover, SEM images show that the least agglomeration and the most homogeneous distribution of Pd NPs were obtained for the sample reduced with ascorbic acid. The lower degree of loading of NPs obtained by the thermal reduction method compared to others was also revealed by SEM analysis, in accordance with the results in Fig. 5a.

The elemental composition of the surface of Pd_Asc@PVP-*g*-PET membrane was investigated by SEM-EDX mapping (Fig. 5c). As can be seen form this EDX mapping, Pd NPs propagate to each point on the PET surface. There are small Pd seeds everywhere, not just in areas where larger particles become apparent as aggregates in SEM images. This is in line with previously reported results^54^ and indicates the presence and distribution of Pd NPs across the entire surface.

**Fig. 6:**
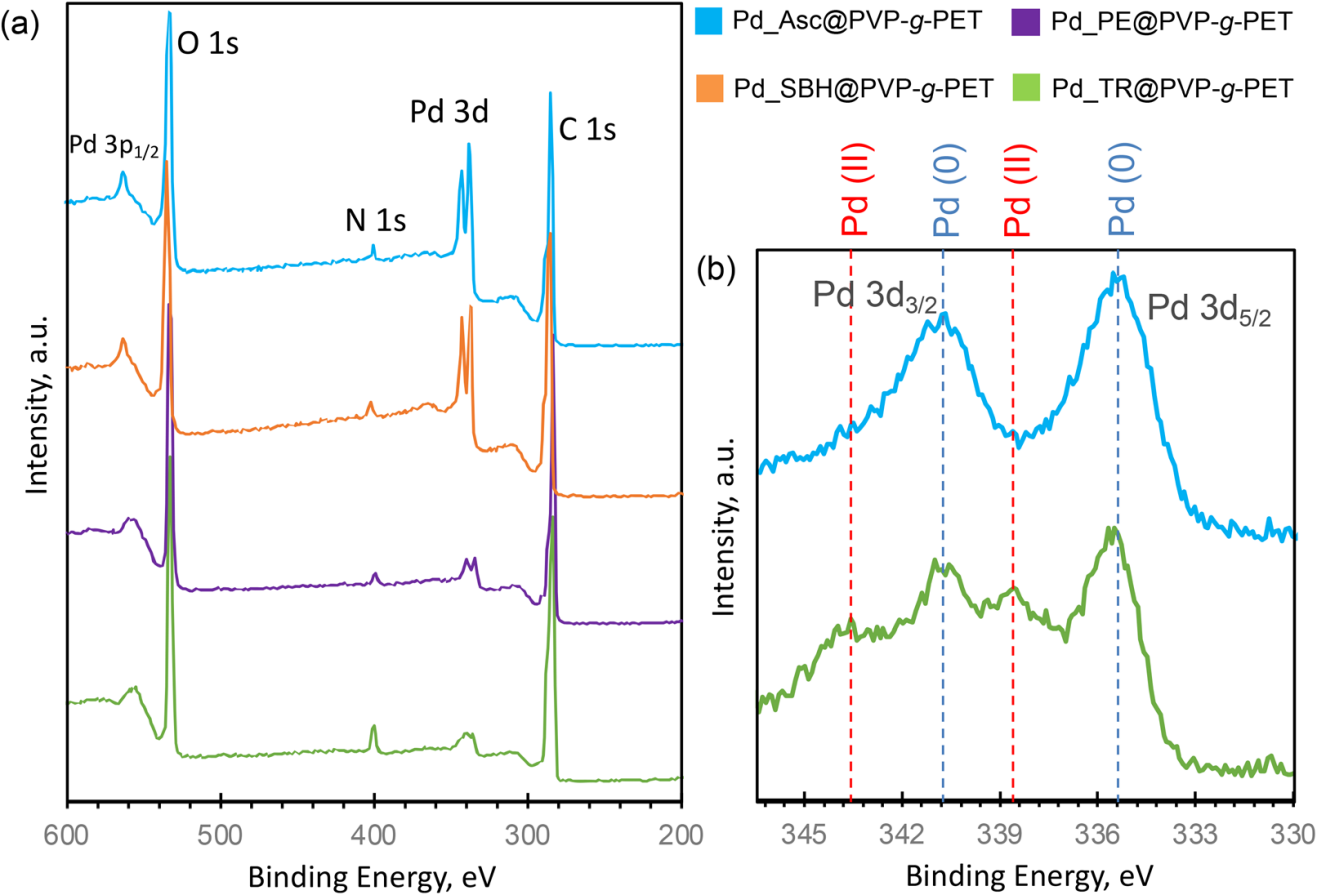
XPS survey wide scans of Pd NPs loaded membranes (a), Pd 3d core level scans of Pd_Asc@PVP-*g*-PET and Pd_TR@PVP-*g*-PET membranes (b).

To further examine the presence and chemical environment of palladium, XPS analyzes of membranes loaded with Pd NPs were performed. As can be seen from Fig. 6a, C and O atoms, which dominate the structures of PET template and PVP, were also predominantly present in the spectra of all samples at around 285 eV and 232.8 eV, respectively. N 1s peak centered at approximately at 400.1 eV confirms the presence of grafted PVP chains in spectra. In addition, Pd 3d and Pd 3P_1/2_ peaks verify the loading of Pd NPs in all membranes.^54^ The intensity Pd 3d peak was significantly higher in the spectra of samples reduced in the presence of Acs and SBH, which is consistent with the Pd NPs loading efficiencies presented in Fig. 5a. To analyze the oxidation step of Pd in detail, core-level Pd scans of the samples reduced by utilizing Asc and thermal treatment were carried out (Fig. 6b). Pd 3d core-level spectra showing the characteristic 3d_5/2_ and 3d_1/2_ components clearly confirmed the presence of Pd element. It is noteworthy in these spectra that while almost completely zero-valence metallic Pd nanoparticles are obtained as a result of reduction with Asc, zero-valence Pd NPs are accompanied by Pd(ii) ions in the thermal reduction method. These results again prove that there is an effective reduction in the presence of Asc.

**Fig. 7:**
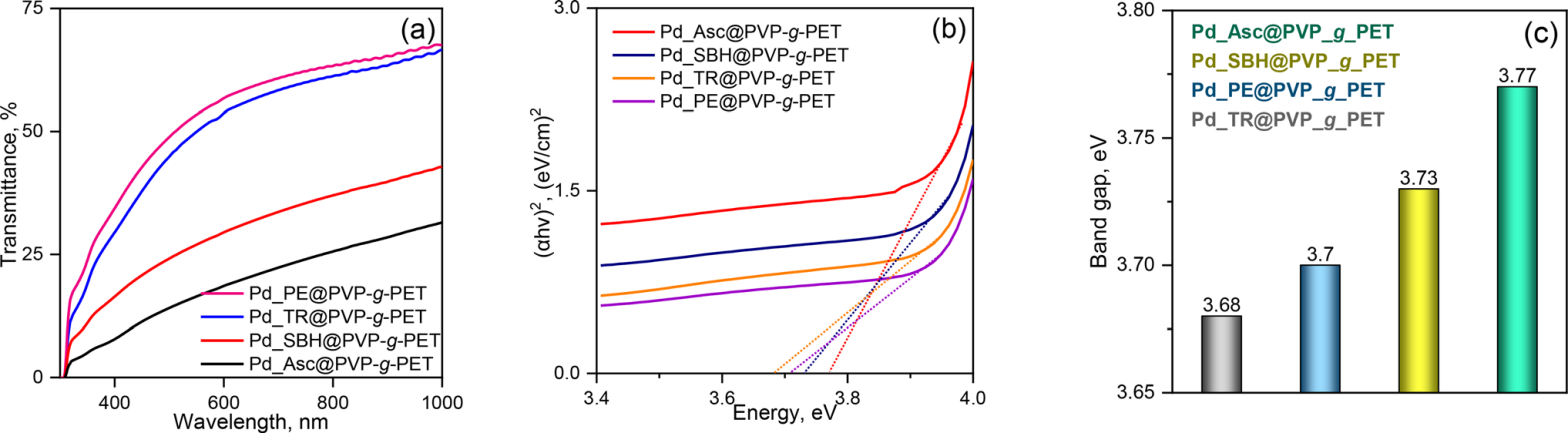
UV-vis transmission spectra (a), Tauc's plot for optical band gap estimation of the composite TeMs (b) and corresponding *E*_g_ values (c).

The optical band gap (*E*_g_) of the materials was estimated using UV-vis spectroscopy. *E*_g_ of the semiconductor materials should be laid between 1.6 and 2.5 eV so that they can absorb light in the visible region of the solar spectrum. Such materials are described as a narrow band gap material, while semiconductor material with wide band gap (∼3.2 eV) can only absorb light in the ultraviolet region. Accordingly, in order to increase the enhancement of conversion of sunlight energy with high efficiency, the absorption range must be extended to involve the regions from visible to infrared.^55^ UV-Vis diffuse reflection spectra of Pd_Asc@PVP-*g*-PET, Pd_SBH@PVP-*g*-PET, Pd_PE@PVP-*g*-PET and Pd_TR@PVP-*g*-PET are presented in the Fig. 7.

Experimentally, the optical absorption coefficient *α* of a semiconductor near the band edge can be expressed by the Tauc plot^56^ as given in the following equation:4
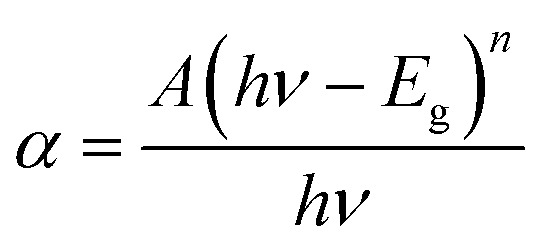
where *α* is the absorption coefficient, *A* is a constant, *E*_g_ is the absorption band, *hν* is the photon energy, and *n* depends on the nature of the transition. That is, *n* can be 1/2, 2, 3/2, and 3 corresponding to the allowed direct, allowed indirect, forbidden direct, and forbidden indirect transitions, respectively. The Tauc plot equation is a commonly used method to analyze the optical absorption coefficient *α* to the photon energy (*hν*). Eqn (4) is sufficient to calculate the band gap energy from the UV-Vis spectra. The change in band energy with different reduction techniques is shown in the inset of Fig. 7. Based on the band gap energies obtained, the calculated band gap for the Pd_Asc@PVP-*g*-PET hybrid catalyst was 3.77 eV. On the other hand, lower values of 3.73 eV, 3.7 eV, and 3.68 eV were determined for the Pd_SBH@PVP-*g*-PET, Pd_PE@PVP-*g*-PET, and Pd_TR@PVP-*g*-PET hybrid catalysts, respectively. In accordance with these results, it can be determined that Pd_Asc@PVP-*g*-PET catalyst indicates the most efficient and rapid catalytic performance in UV-mediated decomposition of MTZ. The highest band gap energy of 3.77 eV for Pd_Asc@PVP-*g*-PET suggests that it requires higher energy for electronic transitions compared to other catalysts with lower band gap energies. This can imply that the Pd_Asc@PVP-*g*-PET hybrid catalyst is more effective in harnessing the energy from UV irradiation to drive the decomposition process. The correlation between the band gap energy and the catalytic performance of the Pd_Asc@PVP-*g*-PET catalyst aligns with previous observations regarding its higher efficiency in rapid degradation of MTZ. Higher band gap energy potentially allows for better utilization of UV energy, resulting in increased generation of reactive species for MTZ degradation.

Comparison of band gap energies among the different catalysts loaded with Pd NPs using various reducing methods can provide insights into the influence of the reduction method on the electronic structure and optical properties of the hybrid catalysts. Band gap energy analysis provides further support for the superior catalytic performance of the Pd_Asc@PVP-*g*-PET membrane in the UV-mediated decomposition of MTZ.

## 4. Catalytic activity results

**Fig. 8:**
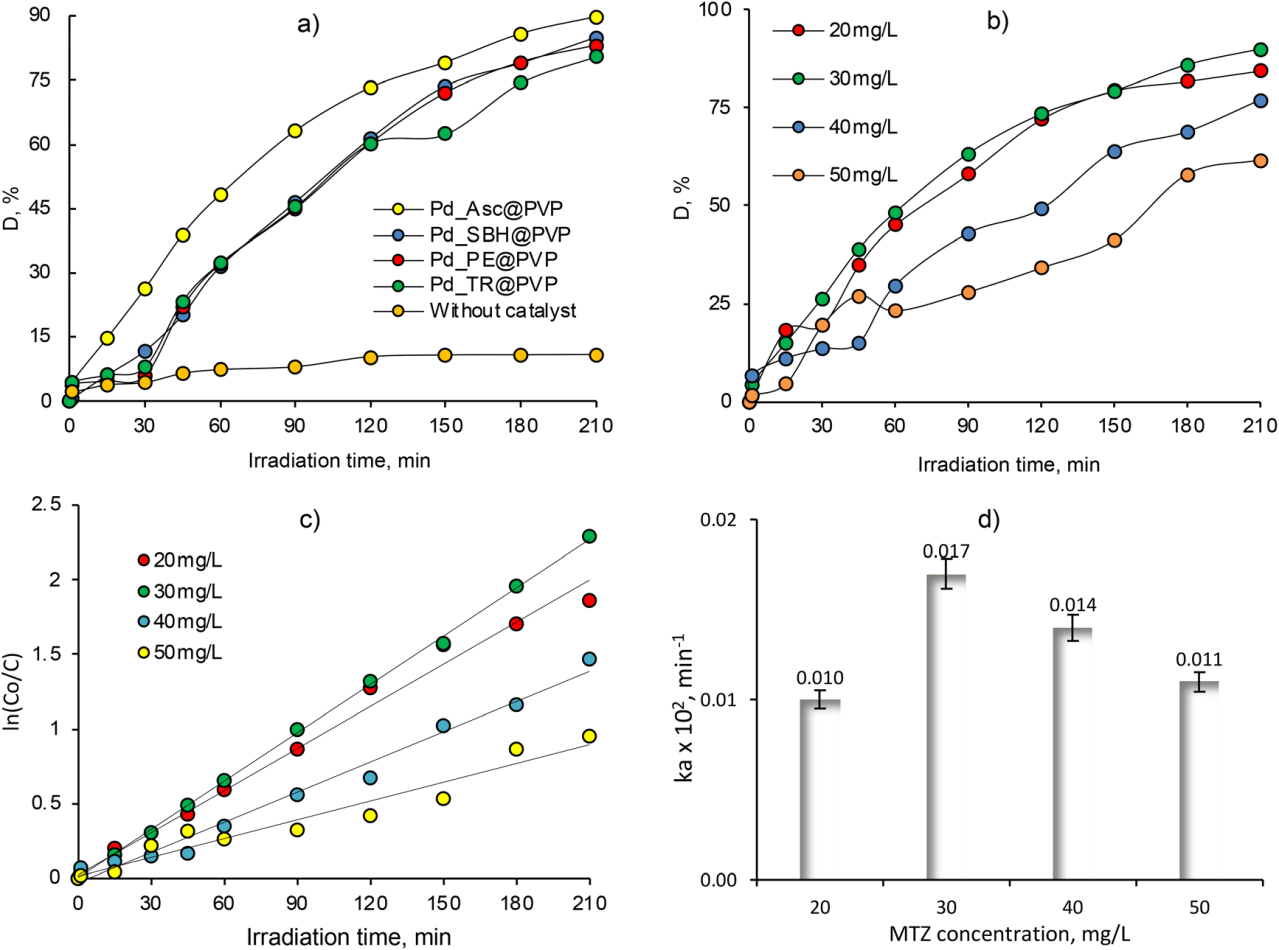
Change in the degree degradation (*D*, %) of MTZ (30 mg L^−1^) depending on the type of catalysts (a), change of degree of MTZ degradation (*D*, %) obtained at various MTZ feed concentrations in the presence of Pd_Asc@PVP-*g*-PET membrane at 30 °C (b), variation of natural logarithm of normalized concentration (ln(*C*_0_/*C*)) as a function of UV-light irradiation time (c) and change of the apparent rate constant *k*_a_ values at different MTZ feed concentrations (d).

Metronidazole (MTZ) is a synthetic antibiotic and antiprotozoal drug widely used to treat a variety of bacterial and parasitic infections.^57^ It works by disrupting the DNA of the microorganisms, causing their death. In this study, the effects of reducing agents, MTX concentration, Pd NPs loading amount, pH, and sorption time on the catalytic activity of composite Pd@PVP-*g*-PET membranes were investigated. The absorption spectra of aqueous MTZ solutions measured in the presence the composite catalyst obtained using different reducing methods are presented in Fig. S2.† After 180 min of exposure to UV-light, the intensity of the characteristic peak of MTZ at 320 nm decreased significantly in the presence of all types of catalysts (Fig. S2a–d†). On the other hand, similar tests performed in the absence of any catalyst membrane did not cause significant changes in absorbance even after 210 min of UV light exposure (Fig. S2e†), and the maximum MTZ degradation (*D*, %) was calculated to be only about 10% as can be seen in Fig. 8a. The degradation degree obtained in the presence of Pd_Asc@PVP-*g*-PET was higher compared to other catalysts and the maximum degree of MTZ degradation was approximately 90%. This catalyst membrane, which was found to be the most effective, namely Pd_Asc@PVP-*g*-PET, was selected as the optimal catalyst and the effect of other parameters on MTX degradation was investigated using this sample.

The effect of initial MTZ concentration on the degradation efficiency under UV-light irradiation was investigated by varying the feed concentration of MTZ solution in the range of 20–50 mg L^−1^. In all experiments, the same amount of catalyst (1 × 1 cm, 21.1 mg of loaded Pd NPs) was employed at a temperature of 30 °C and a pH value of 6.5. Fig. 8b shows the change in the degree of MTZ degradation (*D*, %) under UV-lamp over different sample exposure periods up to 210 min, depending on the MTZ feed concentration. As can be seen, the value of *D* was dependent on the initial MTZ concentration. Almost complete degradation (approximately 90%) occurred in 210 min when the initial concentration of MTZ was 20 mg L^−1^ and 30 mg L^−1^. To calculate the apparent rate constant from the change in the MTZ concentration, the following equation was used:^58^5
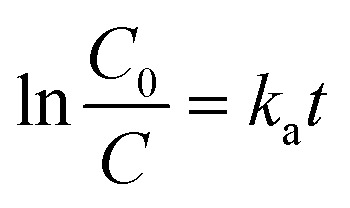
where *C*_0_ is the initial concentration of MTZ (mg L^−1^), *C* is the concentration of MTZ at the irradiation time, *t* (min), and *k*_a_ is the reaction rate constant (min^−1^).

Based on the data obtained, MTZ degradation degree was higher at lower feed concentrations, as expected. As can be seen from the data presented in Fig. 8b and d, when the MTZ concentration is 50 mg L^−1^, the degradation efficiency decreased from its highest value of 89.86% to 61.53%, as well as the apparent rate constant values dropped from 0.017 to 0.011. Quite close results were obtained when the MTZ feed concentration was 20 mg L^−1^ and 30 mg L^−1^, and further experiments were performed at an MTZ feed concentration of 30 mg L^−1^ to examine other parameters. Kinetic curves for UV-induced decomposition of MTZ in the concentration range of 20–50 mg L^−1^ in the presence of Pd/PET hybrid composite (1 × 1 cm) are shown in Fig. 7c. As can be seen from the curves, the Langmuir–Hinshelwood plots were all linear, suggesting that the photodecomposition of MTZ follows the pseudo-first-order reaction kinetics. Accordingly, the calculated *k*_a_ for the 30 mg L^−1^ feed concentration of MTZ was 0.017 × 10^−2^ min^−1^, while lower values of 0.014 × 10^−2^ min^−1^, 0.011 × 10^−2^ min^−1^, and 0.010 × 10^−2^ min^−1^ were established in the presence of 20 mg L^−1^, 40 mg L^−1^, and 50 mg L^−1^ MTZ, respectively. In line with these results, it can be said that in the presence of equal amount of Pd_Asc@PVP-*g*-PET hybrid membrane, UV-mediated decomposition of MTZ is catalyzed more efficiently and rapidly when the feed concentration of MTZ is 30 mg L^−1^. The hybrid membrane combining the Pd NPs with PVP-*g*-PET matrix and utilizing ascorbic acid as the reducing agent was determined as the optimal catalyst for MTZ degradation. This catalyst demonstrated a higher degree of MTX degradation compared to other catalysts, achieving a maximum degradation level of about 90%. Furthermore, the catalytic studies reveal that MTZ exhibits degradation with higher efficiency and rate under UV irradiation when its feed concentration is 30 mg L^−1^, as can be seen in Fig. 8b. Specific mechanisms underlying the enhanced efficiency and rapid degradation may include the effective adsorption of MTZ on the Pd_Asc@PVP-*g*-PET composite, the catalytic activity of Pd NPs in promoting MTZ decomposition, and the synergistic effect between the Pd NPs and the UV irradiation in generating reactive species for MTZ degradation.

**Fig. 9:**
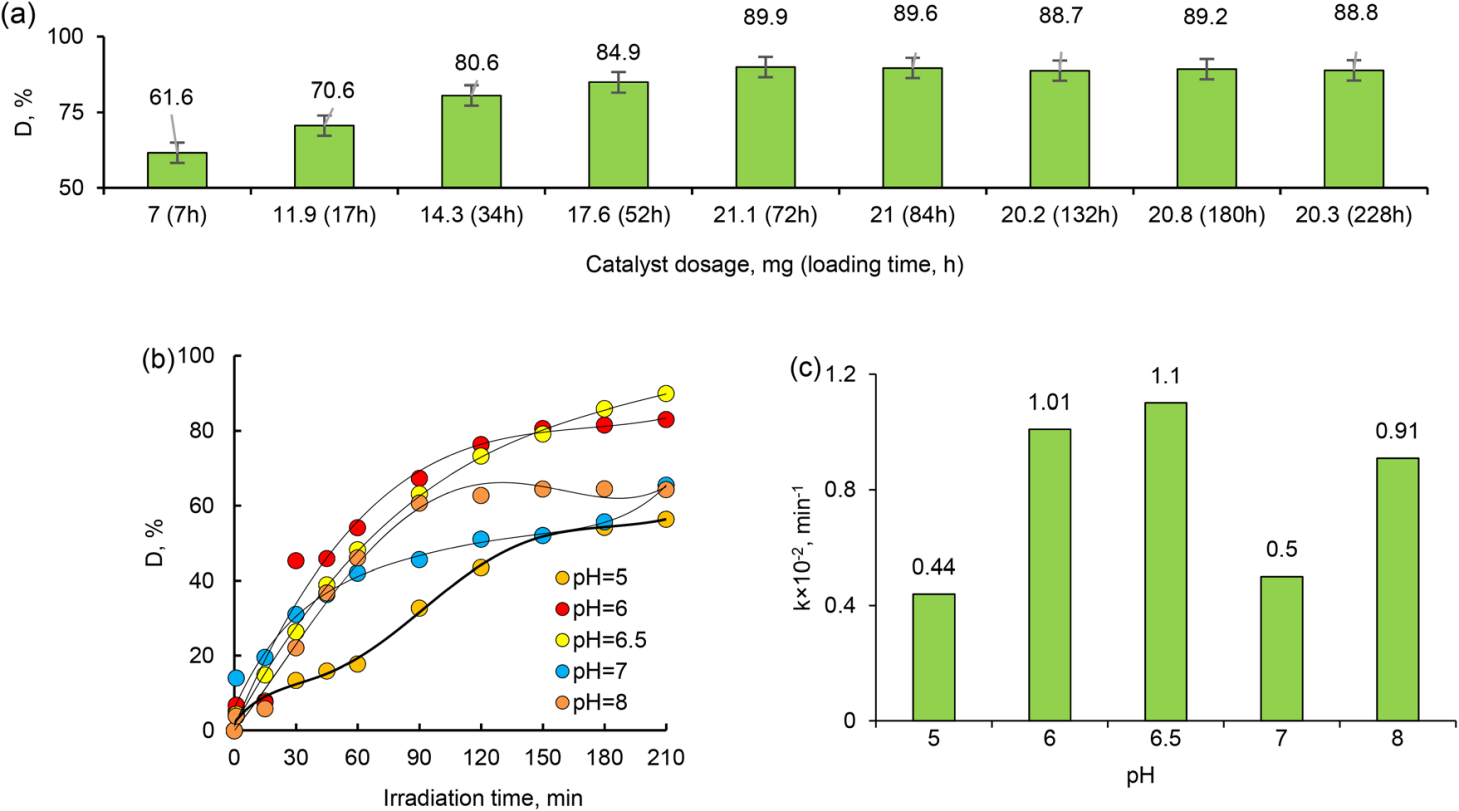
The change of degree of MTZ degradation (*D*, %) achieved at various amount of catalyst in the presence of Pd_Asc@PVP-*g*-PET (a), the change of degree of MTZ degradation (*D*, %) (b) and change of the apparent rate constant *k*_a_ values achieved at various pH values of MTZ solution in the presence of Pd_Asc@PVP-*g*-PET (c).

Catalyst dosage is also an important parameter for optimizing working conditions and comparing the effectiveness of catalysts. Therefore, the effects of the dosage of the active phase (Pd NPs) of the composite catalyst on the degradation of MTZ were investigated at different dosages from 7 mg to 21.1 mg, and the results are given in Fig. 9a. After the 72 hours of loading, there is a reaction equilibrium for degradation of MTZ, as can be seen in Fig. 9a. The results indicated that the amount of degradation gradually increased with increasing catalyst dosage over the studied range. As expected, 21.1 mg catalyst proved the highest photocatalytic degradation efficiency due to the increase in the number of active sites and the high surface area of the composite catalyst. As the catalyst loading increases, more active sites are available to catalyze the degradation reaction. These active sites are typically the catalytic centers, such as the Pd NPs in the Pd_Asc@PVP-*g*-PET composites. Additionally, the high surface area of the composite catalyst contributes to its improved photocatalytic performance. A higher catalyst loading often leads to an increased surface area due to the presence of more nanoparticles. Our finding aligns with the expected behavior and highlights the importance of optimizing catalyst dosage to maximize the performance of photocatalytic activities.

The pH of the solution is one of the key parameters of photocatalytic processes. Changes in pH values affect the surface charge and degree of ionization of the catalyst, the electrostatic interactions between the catalyst surface and the reactant molecules, and the distribution of functional groups in the active centers of the catalyst, as well as the chemical composition of the solution.^59^ To investigate the effect of pH on the degradation efficiency of MTZ, we carried out a series of experiments in the pH range of 4–9, whereby the required pH level was adjusted using 1.0 M HCI_(aq)_ or NaOH_(aq)_. The MTZ concentration was 30 mg L^−1^ and the temperature was 30 °C. As can be seen from the data presented in Fig. 8b, the highest degradation efficiency of MTZ was obtained at the solution's own natural pH of 6.5. When the pH was increased from 5 to 6.5, the maximum removal efficiency increased from 56.35% to 89.86%. However, with a further increase in pH up to 8, the MTZ removal efficiency decreased from 89.86% to 57.82%. Therefore, pH 6.5 was chosen as optimum and further experiments were conducted at this pH.

**Fig. 10:**
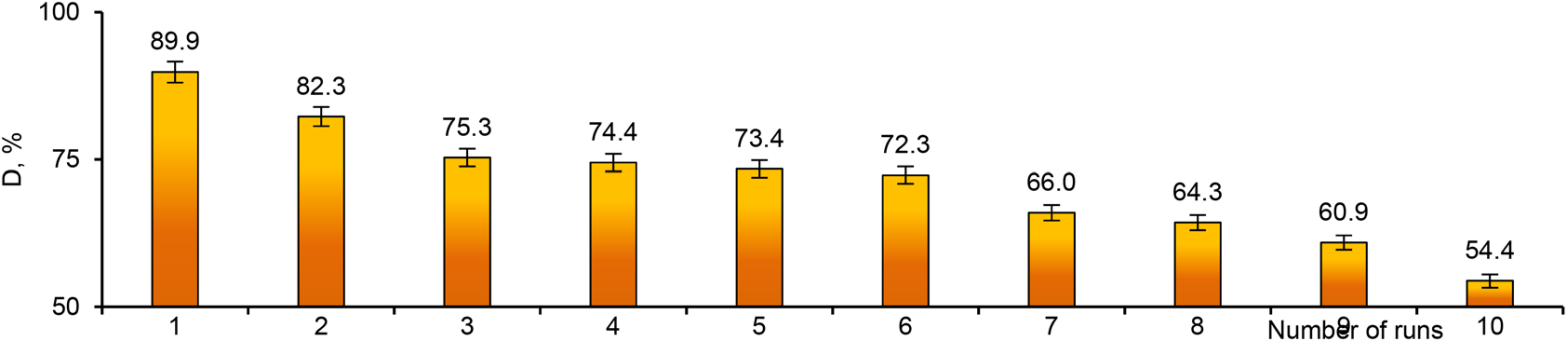
Reusability of the composite catalyst: variation of MTZ degradation degree (*D*, %) with repeated use of Pd_Asc@PVP-*g*-PET catalyst.

Reusability is one of the most important requirements for catalysts. All synthesized composites were tested in several consecutive cycles. As can be seen from the data in Fig. 10, the degradation efficiency decreased gradually after each test cycle. However, even after 10 test cycles, the degradation efficiency of MTZ remained above 50%, which means that the composite catalyst membranes can be used for many consecutive applications. The Pd_Asc@PVP-*g*-PET hybrid catalyst was able to catalyze the degradation of approximately 90% of the pollutant after the 1st cycle, while it was able to remove around 60% and 55%, respectively, in the 9th and 10th cycles. The composite catalyst was able to efficiently degrade MTZ at all runs, demonstrating the excellent recyclability of the Pd NPs-loaded composite track-etched membrane as a catalyst.

Many different types of catalysts for MTZ degradation have been presented in the literature. While these options have gained attention for their potential as catalysts in MTZ degradation, they also have their own limitations that need to be considered. Table 1 presents a comparison of the results obtained in the current study with previously published data on the catalytic activity of various types of catalysts investigated for the degradation of MTZ. However, it should be noted that the direct comparison of data from different studies can be misleading due to differences in experimental conditions such as the amount of loaded catalyst, the initial concentration of MTZ, and the type and size of the catalyst. Despite these challenges, the results obtained in the current study are comparable to existing alternatives, suggesting that the composite membranes developed are promising for practical applications, particularly in terms of ease of preparation and material integrity that can be easily adapted for on-site use. In addition, their high efficacy contributes to ensuring efficient and sustainable degradation of MTZ.

**Table 1:** The catalytic performance of different types of nanosized materials in the degradation of MTZ

Catalyst	Nano-catalyst test conditions						*D*_max_, %	*k*_a_, min^−1^	Reusability of the catalyst, recycling runs	Ref.
	Light source	MTZ, mg L^−1^	Contact time, min	Catalyst dosage, mg	*T*, °C	pH				
TiO_2_ photocatalyst	UV light	50.0	60.0	1500	20.0	6	86.10	—	—	60
ZnO photocatalyst	UV light	30.0	60.0	500	20.0	9.5	60.32	—	—	
ZnO–RGO (reduced graphene oxide) hybrid nanocomposites	Visible light	5.0	160.0	1000	25.0	—	49.3	—	5	61
Nano-ZnO	UV light	80.0	180.0	1500		10	96.55	—	—	62
Niobate K_6_Nb_10.8_O_30_ photocatalyst	UV light	10.0	180.0	1500	25.0	—	57	0.00449	—	63
BiVO_4_/FeVO_4_ heterojunction photocatalyst	Visible light	10.0	180.0	200	25.0	—	91	—	4	64
Ag-doped-Ni_0.5_Zn_0.5_Fe_2_O_4_	UV light	50.0	360.0	10	25.0	3	90.1	0.0098	—	65
Cu_2_S/Ag_2_S/BiVO_4_@a-Al_2_O_3_	UV light	50.0	105.0	1000	25.0	3	96.2	0.0294	6	66
TiO_2_ nanoparticles	UV light	80.0	120.0	500	25.0	7	99.48	0.0233	5	67
Pd_Asc@PVP-*g*-PET	UV light	30.0	180.0	20	30.0	6.5	89.86	0.0118	10	Present study
Pd_SBH@PVP-*g*-PET				14			84.97	0.0085		
Pd_PE@PVP-*g*-PET				7			83.14	0.0082		
Pd_TR@PVP-*g*-PET				4			80.58	0.0073		

## 5. Conclusion

Herein, RAFT-mediated grafting of PVP from the etched nanochannel walls and surface of PET TeMs was achieved *via* the UV-activation of benzophenone. Grafting of PVP to the entire surface provided a functional catalyst for MTZ and stabilized the Pd NPs loaded in the subsequent step. UV-initiated grafting of PVP was studied in aqueous media under various conditions, yielding degrees of grafting ranging from 1.7% to 35.6%. PVP-*g*-PET membranes were characterized by FTIR, XPS, TGA, contact angle and SEM analyses. The SEM results revealed that the polymerization took place from the pore walls and the entire surface of PET TeMs, and the optimal grafting degree at which the nanochannels did not close was around 15%. Grafting degrees exceeding ∼20% were avoided as the nanopores were closed and the mechanical properties deteriorated. SEM-EDX elemental mapping revealed the homogeneity of grafting and loading of Pd NPs. The catalytic degradation of MTZ was investigated in the presence of Pd NPs reduced by different methods under various experimental conditions such as MTZ concentration, pH (5.0–8.0), and Pd NPs loading amount. The reduction of Pd(ii) were carried out using ascorbic acid, sodium borohydride, plant extract, or conventional thermal reduction, and the highest degradation efficiency was obtained in the presence of Pd_Asc@PVP-*g*-PET membrane (89.86% removal for 30 mg per L MTZ) at the natural pH (6.5) of MTZ solution. Optical band gap measurements also showed that the Pd_Asc@PVP-*g*-PET hybrid catalyst was more efficient in harnessing the energy from UV irradiation to drive the decomposition process.

Photodegradation of MTZ was found to follow the pseudo-first-order reaction kinetics. The reaction rate constant was decreased from 0.017 to 0.011 min^−1^ with increasing MTZ concentration from 20 to 50 mg L^−1^, respectively. The photocatalyst revealed respectable photocatalytic activity even after 10 consecutive cycles. Eventually, the composite catalysts, in particularly Pd_Asc@PVP-*g*-PET, were determined as a promising and effective alternative for the removal of antibiotic in an appropriate reaction time.

## Abbreviations

TeM: Track-etched membrane
PET: Poly(ethylene terephthalate)
PVP: Poly(1-vinyl-2-pyrrolidone)
PVP-*g*-PET: Poly(1-vinyl-2-pyrrolidone) grafted PET track-etched membranes
Pd@PVP-*g*-PET: Poly(1-vinyl-2-pyrrolidone) grafted PET track-etched membranes loaded with palladium nanoparticles
Pd_Asc@PVP-*g*-PET: Poly(1-vinyl-2-pyrrolidone) grafted PET track-etched membranes loaded with palladium nanoparticles and reduced with ascorbic acid
Pd_SB@PVP-*g*-PET: Poly(1-vinyl-2-pyrrolidone) grafted PET track-etched membranes loaded with palladium nanoparticles and reduced with sodium borohydride
Pd_PE@PVP-*g*-PET: Poly(1-vinyl-2-pyrrolidone) grafted PET track-etched membranes loaded with palladium nanoparticles and reduced with plant extract
Pd_TR@PVP-*g*-PET: Poly(1-vinyl-2-pyrrolidone) grafted PET track-etched membranes loaded with palladium nanoparticles and reduced with thermal reduction
NPs: Nanoparticles
MTs: Microtubes
RAFT: Reversible addition fragmentation chain transfer polymerization
CPPA: 4-Cyano-4-(phenylcarbonothioylthio) pentanoic acid
MTZ: Metronidazole
BP: Benzophenone
TGA: Thermogravimetric analysis
XRD: X-ray diffraction
XPS: X-ray photoelectron spectroscopy
SEM: Scanning electron microscopy
EDX: Energy dispersive X-ray analysis
CA: Contact angle
DG: Degree of grafting
*D*
: Degradation or degradation (%)
*C*_0_
: Feed MTZ concentration (mg L^−1^)
*C*_e_
: Concentration of MTZ in aliquots (mg L^−1^)
*A*_0_
: Initial absorbance of MTZ solution at 320 nm
*A*
: The absorbance of MTZ at 320 nm at different time intervals
*t*
: Irradiation time (min)
*k*_a_
: Reaction rate constant (min^−1^)

## Author contributions

Conceptualization, A. A. M. and M. V. Z.; methodology, A. A. M., S. D. S and M. B.; validation, P. N., N. A. A. and A. Ya.; formal analysis, A. A. M.; investigation, N. P., A. Ya. and N. A. A.; writing-original draft preparation, P. N., A. A. M., S. D. S. and M. B.; writing-review and editing, P. N., S. D. S., M. B. and A. A. M.; supervision, M. B. and M. V. Z.; project administration, A. A. M.; funding acquisition, A. A. M. All authors have read and agreed to the published version of the manuscript.

## Conflicts of interest

The authors declare no conflict of interest.

## Supplementary Material

RA-013-D3RA03226D-s001
