# Supplementary material for: Environmentally friendly loading of palladium nanoparticles on nanoporous PET track-etched membranes grafted by poly(1-vinyl-2-pyrrolidone) via RAFT polymerization for the photocatalytic degradation of metronidazole
Source: RSC Adv. 2023 Jun 20;13(27):18700–14. doi: 10.1039/d3ra03226d (PMC10281340; doi:10.1039/d3ra03226d)
Supplement: RA-013-D3RA03226D-s001 [file RA-013-D3RA03226D-s001.pdf]

Electronic Supplementary Material (ESI) for RSC Advances.
This journal is © The Royal Society of Chemistry 2023

Supplementary Materials: The following supporting information can be downloaded at:

Environmentally Friendly Loading of Palladium Nanoparticles on Nanoporous PET Track-Etched Membranes grafted by Poly(1-Vinyl-2-Pyrrolidone) via RAFT Polymerization for the Photocatalytic Degradation of Metronidazole

Nursanat Parmanbek[a,b], S. Duygu Sütekin [c,d], Murat Barsbay[c,d], Nurgulim A. Aimanova[a], Anastassiya A. Mashentseva [a,e*], Alisher M. Zhumabayev[a,b], Alyona Yanevich[a], Alimzhan A. Almanov[a,b] and Maxim V. Zdorovets[a,f,g]

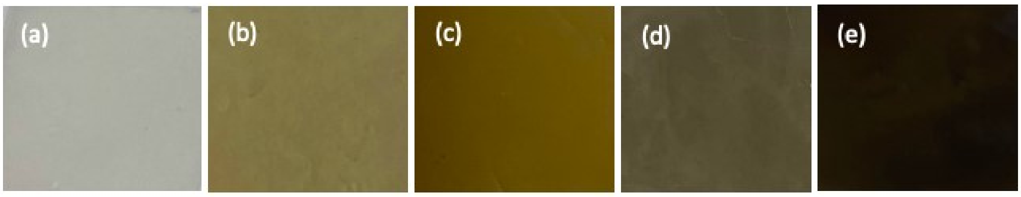

Figure S1 - The color changing tendency of Pd-loaded PVP-grafted samples by varying reducing agents: a) Pristine PVP-g-PET; b) Pd_TR@PVP-g-PET by thermal reduction method; c) Pd_PE@PVP-g-PET by plant extract method; d) Pd_SBH@PVP-g-PET by Sodium borohydride; e) Pd_Asc@PVP-g-PET by ascorbic acid.

[a] *The Institute of Nuclear Physics of the Republic of Kazakhstan, 050032 Almaty, Kazakhstan. E-mail: a.mashentseva@inp.kz*
[b] *Department of Chemistry, L.N. Gumilyov Eurasian National University, 010008 Astana, Kazakhstan.*
[c] *Department of Chemistry, Hacettepe University, 06800 Ankara, Turkey*
[d] *Hacettepe University, Institute of Science, Polymer Science and Technology Division, Beytepe, 06800, Ankara, Turkey*
[e] *Department of Nuclear physics, new materials and technologies, L.N. Gumilyov Eurasian National University, 010008 Astana, Kazakhstan*
[f] *Engineering Profile Laboratory, L.N. Gumilyov Eurasian National University, 010008 Astana, Kazakhstan*
[g] *Department of Intelligent Information Technologies, The Ural Federal University, 620002 Yekaterinburg, Russia*

† Footnotes relating to the title and/or authors should appear here.

Electronic Supplementary Information (ESI) available: [details of any supplementary information available should be included here]. See DOI: 10.1039/x0xx00000x

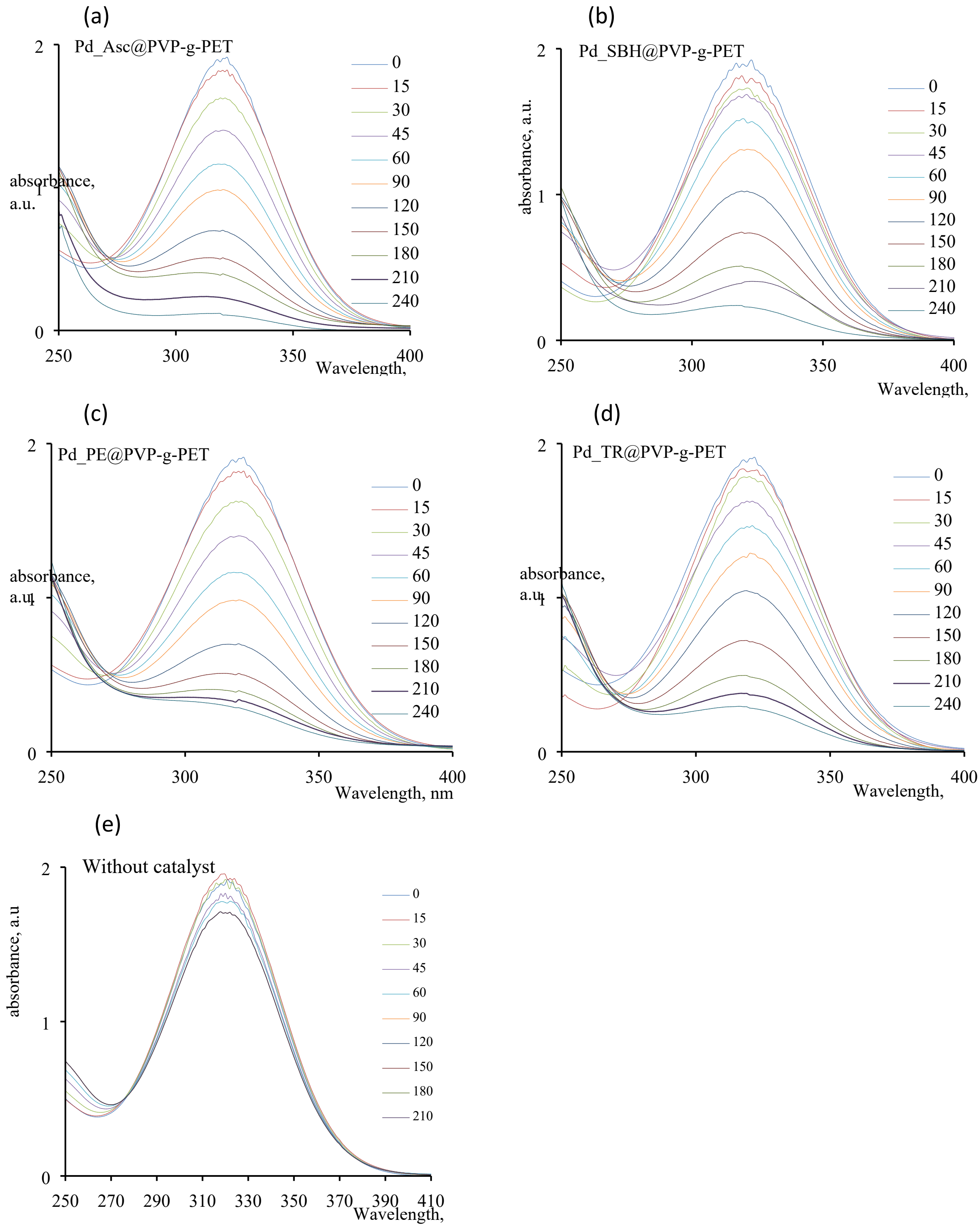

Figure S2 - Typical absorption spectra for the decomposition reaction of MTZ (30mg/l) in the presence of 1x1 cm Pd_Asc@PVP-g-PET (a), Pd_SBH@PVP-g-PET (b), Pd_PE@PVP-g-PET (c), Pd_TR@PVP-g-PET (d) composite membranes and without any catalyst (e).
